# Multi-Objective Optimization and Performance Assessments of an Integrated Energy System Based on Fuel, Wind and Solar Energies

**DOI:** 10.3390/e23040431

**Published:** 2021-04-06

**Authors:** Jingyun Li, Hong Zhao

**Affiliations:** 1 School of Economics and Management, University of Chinese Academy of Sciences, Beijing 100190, China; zhaohong@ucas.ac.cn; 2 Xinjiang Tianfu Jinyang New Energy Co., Ltd., Shihezi 832000, China; 3 Sino-Danish College, University of Chinese Academy of Sciences, Beijing 100190, China

**Keywords:** integrated energy system, gas-wind-photovoltaic system, nondominated sorting genetic algorithm II (NSGA-II), multi-objective optimization, sensitivity analysis

## Abstract

The integrated energy system (IES) is an efficient method for improving the utilization of renewable energy. This paper proposes an IES based on fuel, wind and solar energies, following an optimization study focused on determining optimal device capacities. The study included gas turbines, wind turbines, solar photovoltaic panels, ground source heat pumps, absorption chillers/heaters, batteries, and thermal storage. Objectives were incorporated into the optimization model for the overall performance of the IES; these included the primary energy saving rate, annual cost-saving rate, and carbon dioxide emission reduction. Then, the nondominated sorting genetic algorithm II was employed to solve the optimization problem for multiple objectives. Ultimately, the verification and sensitivity analyses of the optimization method were achieved by a case study of hospital buildings in Harbin. The optimization results indicated a primary energy saving rate, annual cost saving rate, and carbon dioxide emission reduction rate of 17.3%, 39.8%, and 53.8%, respectively. The total installed capacity for renewable energy generation accounted for 64.5% of performance optimization. Moreover, the price of natural gas affected the economic indicators of the IES–but failed to impact energy consumption indicators.

## 1. Introduction

Promoting the transformation of energy structures and improving the efficiency of energy utilization are urgent issues, given the increasing rate of consumption of coal, oil and other traditional energy sources [1]. Fortunately, the promotion and application of integrated energy systems (IES) can effectively improve the utilization rate of renewable energy sources, and actively promote the preservation of the environment [2]. However, complex equipment capacity configurations and various system operation strategies limit the further development of IES.

Optimization is one of the most effective methods to solve these problems [3]. Many researchers have concentrated on the problem of IES systems through various optimization goals, which are typically classified into single and multiple objective goals. Wei et al. [4] established a set of integrated day-ahead coordination and optimization operation models for multi energy power systems (MEPS) with economy, safety and renewable energy consumption maximization as multiple optimization objectives. To realize sustainable development of an IES, Wang et al. [5] extended the concept of demand response (DR) and established a multi-objective optimization model with the goals of economic profit and energy efficiency for the first time. By establishing different models, the results showed that model 3 reduced costs by 3.74% compared with model 1. Model 1 considered using time-price, while in model 3, users participated in DR schemes incentivized by real-time price and DR compensation. In that model, CO_2_, SO_2_ and NOx emissions were reduced by 0.26 kg, 0.45 kg and 0.05 kg, respectively.

Improving the utilization rate of energy has always been the core objective of optimization. Hu et al. [6] used the energy quality coefficient (which measures the quality of various forms of energy) to evaluate energy. The multi-objective programming model—with energy efficiency and economy as its objectives—was established. Emphasis was placed on transforming nonconvex problems into convex problems. In addition, in order to improve the comprehensive utilization efficiency of various types of energy and obtain more economic benefits, the accurate prediction of various loads in the comprehensive energy system must be considered key. Tan et al. [7] established various load combination prediction models based on multitask learning and least-squares support vector machines. The results showed that the average prediction accuracy of the model was improved by 18.60% in comparison with extreme learning.

Optimization problems can be divided into linear programming, nonlinear programming and dynamic programming. In order to solve these problems, optimization methods—including classical methods and artificial intelligence algorithms—were proposed and developed. The structure, system scale and performance of integrated energy systems were improved using the genetic algorithm [8], particle swarm optimization [9] and artificial neural networks [10]. IES optimization research mainly focuses on system integration design and operation management. The optimal energy output of the equipment was obtained by using the fly optimization algorithm [11] with poor convergence. The multi-objective particle swarm optimization [12] was used to find the appropriate capacity and position of each component and increase system dependability. The genetic algorithm [13] was used to realize the hourly optimal scheduling method of system components and reduce the daily operation cost. The nondominated sorting genetic algorithm II (NSGA-II) [14] was used to determine the appropriate component size, and the system performance was greatly improved through reasonable operation management. As highlighted in [15], multi-objective optimization is the process of finding as many Pareto solutions as possible. NSGA-II can indeed find a diversified solution set, and select the optimal solution according to subjective objective requirements. Converting the IES optimization problem into mixed-integer quadratic programming can effectively simulate the dynamic load and the randomness of the load. The strength Pareto evolutionary algorithm was also stronger than NSGA in random simulation. Detailed comparisons of different algorithms are listed in Table 1.

The optimal IES usually operates under uncertain conditions, through system design [17]. Liu et al. [18] introduced subjective and cognitive uncertainties into the integrated demand response (IDR) model based on price—and introduced evidence theory and credibility levels to handle the double uncertainties. The final results showed that the risk of system operation could be reduced by considering the uncertainty of IDR, but the cost increased slightly. Su et al. [19] established a two-stage optimization model to examine the ability of an IES to meet energy demands under the uncertainty of coupling renewable energy demand and operation. In view of the uncertainty of wind power, Turk et al. [20] proposed a two-stage random scheduling scheme for IES, and a practical case was given to prove the economy and the scheme’s improvements in wind power utilization efficiency. Mohammadi et al. [21] used the fuzzy set method to study the uncertain modeling problem which affects the energy hub operation (for the first time). The optimization studies showed that the optimal operation cost of the scheme was effective when the membership degree was 0.412. At that time, the system cost increased by 2.9% under uncertainty, but the accuracy and reliability of the model were greatly improved.

This paper studied the optimization problem of an IES involving wind, solar, natural gas cogeneration and multi-energy complementary power grid. The researchers evaluated the IES on energy efficiency, economic operation and preservation of the environment using the primary energy saving rate, annual cost-saving rate and carbon dioxide emission reduction rate.

The innovation of this article lies in the following aspects:

1. Use multi-objective optimization algorithms to evaluate system performance.
2. Provide efficient system operation plan through energy dispatch analysis.
3. Conduct sensitivity analysis on main parameters to deal with changes in energy fuel prices.

The structure of this paper is as bellow: Section 2 introduces the structure and operation strategy of the IES. Section 3 presents optimized objects and solution algorithms. Section 4 delivers the conclusions.

## 2. Gas-Wind-Photovoltaic Integrated Energy System

### 2.1. Modelling

**Figure 1:**
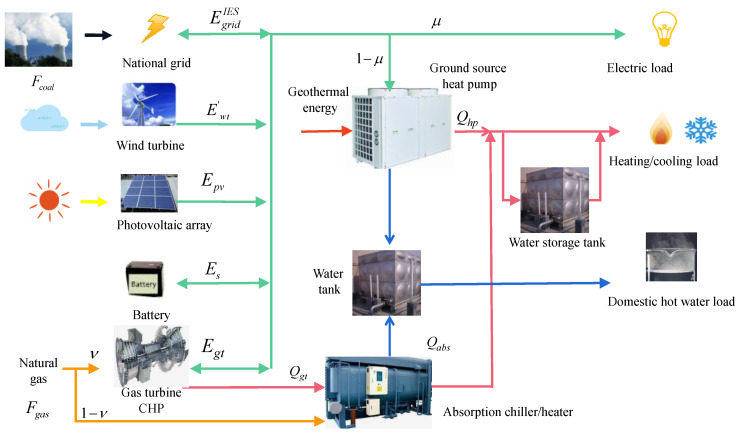
Energy flows of an integrated energy system.

Figure 1 describes the energy flow of the IES presented in this paper. There are several main energy inputs, including: natural gas, solar, wind, coal, and geothermal energy. The energy demands include electricity, heating, cooling, and domestic hot water. IES consists of an electric grid, wind turbine (WT), gas turbine (GT), combined heat and power (CHP) system, photovoltaic array (PV), ground source heat pump (GSHP), absorption chiller/heater (AC/H), water storage tank for heat/cold energy and battery for electricity storage.

Power demand is mainly provided by the WT, PV, and GT to meet building consumer needs—along with a GSHP for cooling or heating. When the electricity generated is insufficient, it is assisted by the electric grid. Otherwise, the remaining electricity from the WT, PV, and GT is returned to the electric grid. The role of the battery in the IES is to store excess electricity or supplement insufficient electricity.

Chilled and hot water are produced by the absorption cycle driven by the waste heat of the high-temperature flue gases. The GSHP consumes electricity to generate chilled water or heating water required by building loads—which effectively balances the heat and electricity ratio between the system and users. If the waste heat cannot meet the heat demand, natural gas is directly burned into the AC/H to supplement heat; otherwise, the excess heat can be stored in the water storage tank to supplement the insufficient heat. The grid, water storage tank and battery also serve as auxiliary equipment to store excess energy or compensate for deficiencies, further increasing the flexibility and stability of the system.

#### 2.1.1. Coal Power Plant with National Grid

The electricity provided to buildings is determined by the amount of coal consumed by the national grid, $E_{gird}^{IES}$ (kW), and can be expressed as Equation (1)

(1) $$E_{grid}^{IES}(t)=\eta_{ce}F_{coal}(t),\forall \;t\in T$$

where $F_{coal}$ (kW) is the coal consumption of the power plant; $\eta_{ce}$ is power generation efficiency.

#### 2.1.2. Wind Turbine

Wind speed restricts the power generation of the WT in real time; however, the power output of the WT usually approximately satisfies Weibull distribution, and its power output can be expressed as [22]:

(2) $$E^{\prime }{}_{wt}(t)=\left\{ \begin{array}{ll} E^{\prime }{}_{wt}(t)=0, & V<V_{ci}\\ E^{\prime }{}_{wt}(t)=aV^{3}-bE_{r}, & V_{ci}<V<V_{co}\\ E^{\prime }{}_{wt}(t)=P_{r}, & V_{r}<V<V_{co}\\ E^{\prime }{}_{wt}(t)=0, & V_{co}<V \end{array}\right.$$

where the ratios represented by *a* and *b* are defined as: $a=\frac{P_{r}}{V_{r}^{3}-V_{{}_{ci}}^{3}}$, $b=\frac{V_{{}_{ci}}^{3}}{V_{r}^{3}-V_{{}_{ci}}^{3}}$, respectively; $V_{r}$, $V_{ci}$, $V_{co}$ and $P_{r}$ denote the rated wind speed, cut-in wind speed, cut-off wind speed and rated power of the wind turbine, respectively.

Total power generation from the WT is calculated according to [22]:

(3) $$E_{wt}(t)=E^{\prime }{}_{wt}(t)A_{W}N_{W}\eta_{W\text{-}inv}$$

where $A_{W}$ is the swept area of the WT; $N_{W}$ denotes the number of WT installed; and $\eta_{W\text{-}inv}$ is the inverter efficiency.

#### 2.1.3. Gas Turbine CHP

The power generated by the GT, $E_{gt}$ (kW), is expressed as:

(4) $$E_{gt}(t)=\nu \eta_{gt}^{e}F_{gas}(t),\forall \;t\in T$$

and the waste heat of the exhaust gas from the GT, $Q_{gt}$ (kW), is estimated as:

(5) $$Q_{gt}(t)=\nu \eta_{gt}^{h}F_{gas}(t),\forall \;t\in T$$

where $F_{gas}$ (kW) is the natural gas consumption, $\eta_{gt}^{e}$ and $\eta_{gt}^{h}$ are the power generation and waste heat conversion efficiency, respectively, and the factor ν is used to express the ratio of $F_{gas}$ consumed by the GT to the total consumption.

#### 2.1.4. PV

The power generation from the PV is related to ambient temperature and solar radiation intensity, and the power generation, $E_{PV}$, is expressed as [23]:

(6) $$E_{pv}=fN_{pv}\left[\frac{G_{p}}{G_{stc}}\right]\left[1+\alpha \left(T_{pv,p}-T_{pv,stc}\right)\right]$$

where f is power reduction factor caused by air fouling and material physical properties change; $N_{pv}$ (kW) is the equipment capacity of PV; $G_{p}$ (kW/m^2^) is the solar radiation intensity; $G_{stc}$ (1 kW/m^2^) is the solar radiation intensity under standard test conditions; α (%/°C) is the temperature coefficient; $T_{pv,p}$ (°C) is the PV surface temperature; $T_{pv,stc}$ (25 °C) is the PV temperature under standard test conditions. In addition, $T_{pv,stc}$ can be calculated by the following formula [23]:

(7) $$T_{pv,p}=\frac{T_{a,p}+\left(T_{pv,soc}-T_{a,soc}\right)\left(\frac{G_{p}}{G_{soc}}\right)\left[1-\frac{\eta_{e,pv}\left(1-\alpha T_{pv,stc}\right)}{\tau \beta }\right]}{1+\left(T_{pv,soc}-T_{a,soc}\right)\left(\frac{G_{p}}{G_{soc}}\right)\left(\frac{\alpha \eta_{e,pv}}{\tau \beta }\right)}$$

where $T_{a,p}$ (°C) is the ambient temperature; $T_{pv,soc}$ is the PV surface temperature under standard operating conditions (45~48 °C); $\eta_{e,pv}$ is the PV efficiency under standard test conditions; τ is the transmittance of solar energy; β is the solar energy absorptivity of PV; the default value of τβ is 0.9; standard operating conditions are standard light intensity of ($G_{soc}$) 0.8 kW/m^2^, and standard ambient temperature $\left(T_{a,soc}\right)$ of 20 °C.

#### 2.1.5. Absorption Chiller/Heater

The temperature of cooling water from the PV/T collector is much lower than that of the exhaust gas. The double-effect absorption chiller/heater is driven by waste heat from the GT, and natural gas is employed to make full use of waste heat. Its general output ($Q_{abs}$) of heating, $Q_{h,abs}$ (kW), and cooling, $Q_{c,abs}$ (kW), is expressed as:

(8) $$Q_{abs}(t)=COP_{abs}\left[Q_{gt}(t)+(1-\nu )\eta_{dcz}F_{gas}(t)\right],\forall \;t\in T$$

where $COP_{abs}$ is the coefficient of performance (COP). The energy flows in the cooling and heating modes are different, and their *COP* values are different. The *COP* in cooling mode, $COP_{abs}^{c}$, is determined by the heat ratio of the low-pressure generator ($Q_{LG}$) to the high-pressure generator ($Q_{HG}$) in the absorption chiller, and its coefficient—obtained by simulation with engineering equation solver (EES) [24]—can be fitted to:

(9) $$COP_{abs}^{c}=0.0251{\left(\frac{Q_{LG}}{Q_{HG}}\right)}^{2}-0.2158\frac{Q_{LG}}{Q_{HG}}+1.4058$$

The absorption chiller/heater serves as a heater in heating mode, and its COP, $COP_{abs}^{h}$, is assumed to be 0.9. In addition, it will also output domestic hot water (in both cooling and heating modes) through heat exchange in the low-pressure generator.

#### 2.1.6. GSHP

The output of heat energy, $Q_{h,hp}$ (kW), or cold energy, $Q_{c,hp}$ (kW), from the GSHP is determined by the inputted electric and geothermal energies, and it is generally expressed as:

(10) $$Q_{hp}(t)=f\left(E_{gird}^{IES}(t),Q_{geo}(t)\right)=(1-\mu )COP_{hp}E_{gird}^{IES}(t),\forall \;t\in T$$

where $COP_{hp}$ is the COP of the GSHP; the factor μ is the ratio of $E_{gird}^{IES}$ consumed by the buildings, and 1−μ is the ratio consumed ratio by the GSHP.

#### 2.1.7. Energy Storage System

The energy conversion status of the battery is as follows:

(11) $$E_{s}^{1}=E_{s}^{0}(1-\eta_{s,los})+(\xi E_{s,in}\eta_{s,ch}-(1-\xi )E_{s,out}/\eta_{s,disch})\Delta t$$

(12) $$E_{s}^{\min}\leq E_{s}\leq E_{s}^{\max}$$

(13) $$0\leq E_{s,in}\leq E_{s,in}^{\max}$$

(14) $$0\leq E_{s,out}\leq E_{s,out}^{\max}$$

where $E_{s,out}$ and $E_{s,in}$ (kW) are the discharge capacity and charge capacity of the battery, respectively; $E_{s}^{0}$ and $E_{s}^{1}$ (kW) are the energy storage state of the battery before and after charging/discharging; $\eta_{s,los}$,$\eta_{s,ch}$ and $\eta_{s,disch}$ are the self-consumption rate, charging efficiency and discharging efficiency of the battery, respectively; ξ value is 0 or 1; The time interval Δt is 1 h; $E_{s}^{\max}$, $E_{s}^{\min}$,$E_{s,in}^{\max}$ and $E_{s,out}^{\max}$ (kW) are the maximum and minimum energy storage, maximum charging capacity and maximum discharge capacity of the battery, respectively; These values can be obtained by multiplying the rated capacity of the battery by the corresponding coefficient.

The model of the water storage tank can be expressed as:

(15) $$Q_{h,wst}^{1}=\eta_{wst}Q_{h,wst}^{0}+Q_{h,wst,in}-Q_{h,wst,out}$$

where $Q_{h,wst}^{0}$ and $Q_{h,wst}^{1}$ (kW) are the energy storage state of the storage tank before and after heat storage/release, respectively; $Q_{h,wst,in}$ and $Q_{h,wst,out}$ (kW) are the storage and release heat of the storage tank, respectively; $\eta_{wst}$ is the thermal efficiency of the storage tank.

### 2.2. Operation Strategy

The operation strategy of following electric load (FEL) was adopted; the key operational strategy of FEL is to give priority to PV, WT and battery, such that the GT generates no excess electricity. When the heat produced by the gas turbine exceeds the heating demand, the excess heat is stored in the storage tank or directly discharged into the environment; when the heat produced by the gas turbine is less than the heat load, the direct combustion zone of AC/H or the water storage tank makes up for the deficiency. In addition, due to the existence of PV and WT (whose outputs are uncertain), it is necessary to introduce batteries for adjustment. Detailed operating situations include the following cases:

**Case 1.** If $E_{u}+E_{hp}^{IES}<E_{PV}+E_{wt}+E_{s,out}+E_{\min}$, where $E_{hp}^{IES}$ is the inputted electric and $E_{\min}$ (kW) is the minimum power generation of the GT, at the same time, the GT will not operate. There are three potential versions of this situation: (1) $E_{u}+E_{hp}^{IES}<E_{PV}+E_{wt}$: the power required is met by only the PV and WT, and the excess PV and WT electricity will be stored in the storage cell or sold to the national grid. (2) $E_{PV}+E_{wt}<E_{u}+E_{hp}^{IES}\leq E_{PV}+E_{wt}+E_{s,out}$: the demand for electricity is met by PV, WT, and storage battery. (3) $E_{PV}+E_{wt}+E_{s,\mathrm{out}}<E_{u}+E_{hp}^{IES}\leq E_{PV}+E_{wt}+E_{s,out}+E_{\min}$: the power required is provided by PV, WT, battery, and grid.

**Case 2.** When $E_{PV}+E_{wt}+E_{s,out}+E_{\min}\leq E_{u}+E_{hp}^{IES}<E_{PV}+E_{wt}+E_{s,out}+E_{\max}$, where $E_{\max}$ (kW) is the maximum power generation of the GT. The GT operates under partial load to meet the electric load and generates a certain amount of heat. When the heat generated exceeds the heating requirements, the excess heat is released directly into the environment; when the heat generated is less than the heat required, the natural gas direct combustion zone of the AC/H will supply supplementary heat.

**Case 3.** When $E_{PV}+E_{wt}+E_{s,out}+E_{\max}\leq E_{u}+E_{hp}^{IES}$, the GT operates at rated conditions, generating heat and electricity, and supplementary electricity is purchased from the grid. The excess heat is released directly into the ambient atmosphere when the heat produced exceeds requirements; when the heat generated is not enough to meet the load demand, the direct combustion zone of AC/H directly burns natural gas to supplement the remaining driving heat.

## 3. Methodology

### 3.1. Optimization Objectives

The primary energy saving rate (PESR), annual cost saving rate (ACSR) and carbon dioxide emission reduction rate (CDERR) are usually used to assess (respectively) the energy, economic and environmental efficiencies of an IES in comparison to traditional systems. They are (respectively) defined as follows [23]:

(16) $$PESR=\frac{\sum_{t=1}^{o}F_{}^{ref}(t)-\sum_{t=1}^{o}F_{}^{IES}(t)}{\sum_{t=1}^{o}F_{}^{ref}(t)}\times 100\%$$

(17) $$ACSR=\frac{AC^{ref}-AC^{IES}}{AC^{ref}}\times 100\%$$

(18) $$CDERR=\frac{\sum_{t=1}^{o}CDE_{}^{ref}(t)-\sum_{t=1}^{o}CDE_{}^{IES}(t)}{\sum_{t=1}^{o}CDE_{}^{ref}(t)}\times 100\%$$

where ο represents annual operating hours (h), and $F^{ref}$ and $F^{IES}$ (kW) are the fuel consumption of the reference system and IES, respectively. $AC^{ref}$ and $AC^{IES}$ ($) represent the annual costs of the reference system and the IES, respectively. $CDE^{ref}$ and $CDE^{IES}$ are carbon dioxide emissions from the reference system and the IES, respectively. 

### 3.2. Decision Variables

The decision variables in the IES can be divided into design variables and operational variables. The key components’ independent sizing decision variables are as follows: 

Capacity sizes of the GT ($N_{gt}$), PV panels ($N_{PV}$) and wind turbines ($N_{w}$). In the IES, the GT is a core component which affects the capacity of other equipment. The introduction of solar energy and wind energy reduces fuel consumption and carbon dioxide emission, improving the environmental protection of the system, but also increasing the AC of the system.

Capacities of energy storage devices, including battery and water storage tank, are defined as ($N_{s}$ and $N_{wst}$). Energy storage devices and WSTs with sufficient capacities are beneficial; they improve the flexibility and stability of the IES, and their capacities can adjust the economic and energetic performances of the system.

An additional operational variable of the IES is the heating or cooling output ratio of the GSHP. The GSHP and AC/H are used to satisfy the users’ space heating or cooling requirements; the operational ratio of the GSHP (θ) refers to the hourly ratio of cooling/heating supplied by the GSHP to the total cooling/heating output.

The optimization variables involved in the IES are as follows:

(19) $$X={\left[N_{gt},N_{pv},N_{wt},N_{s},N_{wst},\theta \right]}^{T}$$

The optimization model considers the maximization of multi-objectives such as PESR, ACSR and CDERR to improve energy, economic and environmental performance respectively. The optimization problem is expressed as:

(20) $$\begin{array}{l} \left\{ \begin{array}{l} \mathrm{Max}\;PESR(X)\\ \mathrm{Max}\;ACSR(X)\\ \mathrm{Max}\;CDERR(X) \end{array}\right.,X\in R^{n}\\ S.t.\;h_{i}(X)=0,\;(i=1,2,\cdots ,p)\\ \;\;g_{i}(X)\leq 0,\;(i=p+1,p+2,\cdots ,q) \end{array}$$

where $h_{i}(X)$ and $g_{i}(X)$ list the restrictions of equalities and inequalities with the decision variables *X* in the *n* dimensional space $R^{n}$, and the functions are subject to these constraints.

### 3.3. Solution Algorithm

This paper aimed—through the operation of the components—to obtain the optimal capacities and thus the most efficient performance of the IES. In the problem of multi-objective optimization, it is hard to get optimal solutions for each objective function at the same time. Therefore, we used the global search capability of the genetic algorithm to avoid traditional optimization methods, falling into the optimal local solution in the optimization process. The optimization problem had a set of Pareto optimal solutions selected by subjective weights.

**Figure 2:**
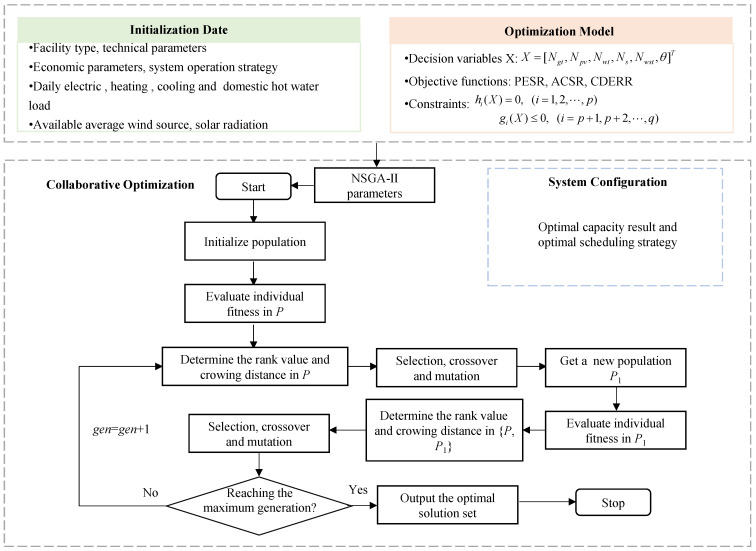
Flowchart of the optimization algorithm.

NSGA-II had incomparable advantages in multi-objective optimization, which combined the parent scheme with its offspring schemes and competed together to produce the next generation population, retaining the diversity of solutions. The decision variables included device decision variables and operating decision variables. When considering discrete variables, the use of mixed integer linear programming [25] or linear programming solvers [26] introduces binary variables and ordinal optimization variables, which increases the computational complexity. Considering that the optimal device variable is a continuous variable, NSGA-II was used to solve the problem, to reduce the computational complexity. As shown in Figure 2, the main calculation process for finding the best result included the following steps:

Step 1: Input the initial parameters. Initial parameters include system integration parameters and NSGA-II setting parameters. System parameters are mainly based on facility type, technical parameters, economic parameters, system operation strategy, etc., and are used to establish system thermodynamic, economic, and emission models. The NSGA-II parameters include the size of population (pop), number of iterations (gen), crossover’s probabilities and mutation ($P_{c}$ and $P_{m}$), and distribution indices of crossover and mutation operations (*mu* and *mum*).

Step 2: Initialize the population. Based on the initialization in Step 1, the *Y* group of decision variables in Equation (4) is randomly generated, where *Y* is the overall size and *P* represents the population.

Step3: According to the operation strategy and objective function, calculate the individual fitness function.

Step 4: Reserve some of the candidate solutions according to fitness in a new population, P1, and discard others.

Step 5: Crossover and mutation operations. A new population, P2, is obtained by crossover and mutation operation of population P1. Then, calculate the individual fitness function in the population P2.

Step 6: Generate a new population. The new population, P, is acquired from {P, P2}, considering the rank value and crowding distance.

Step 7: Termination condition judgment. When the maximum evolutionary generation is contented, the individual with the maximum fitness obtained in the evolution process is taken as the optimal solution output, and the calculation is terminated.

## 4. Results and Discussions

The case study in this paper was aimed at the particularity of the climate in severely cold areas, and carried out related research on reducing environmental pollution, improving energy efficiency, and saving economic investment. Harbin is a severely cold region in China, and experiences harsh weather. To verify the correctness and effectiveness of the system model and optimization method, the hospital buildings in Harbin were selected as a case study. In this case, the regional buildings were supplied with energy by an IES and separate supply (SP) system, respectively. The performance assessments of the IES were evaluated by the optimization results. The optimization process was calculated by MATLAB R2019a software produced by MathWorks company of Massachusetts in the United States, using a Lenovo notebook computer with Intel Core i7-1065G7.

### 4.1. Initializations

**Figure 3:**
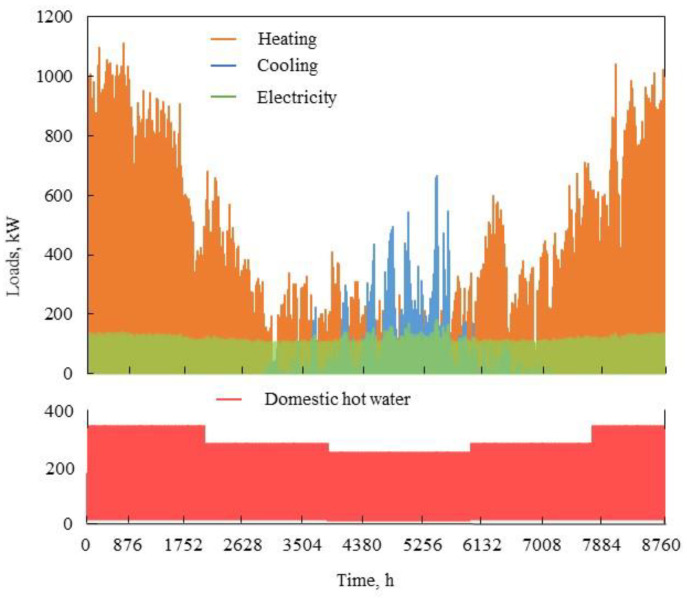
Simulation data of the annual cooling, heating, electric and domestic hot water load of the Harbin hospital.

Figure 3 shows the load simulation data (calculated by DeST software) of a certain Harbin hospital in a year. Because the hospital load is a special type of building load, there is no strict sense of cooling season or transition season. Compared with other buildings, hospitals have more variable load conditions. For example, on a certain day in summer, a demand for both cooling and heating loads may be required, because the hospital requires accurate temperature control. In a year, hospitals experience almost all the demands of heating load and domestic hot water load, while the cooling load is larger in summer.

**Figure 4:**
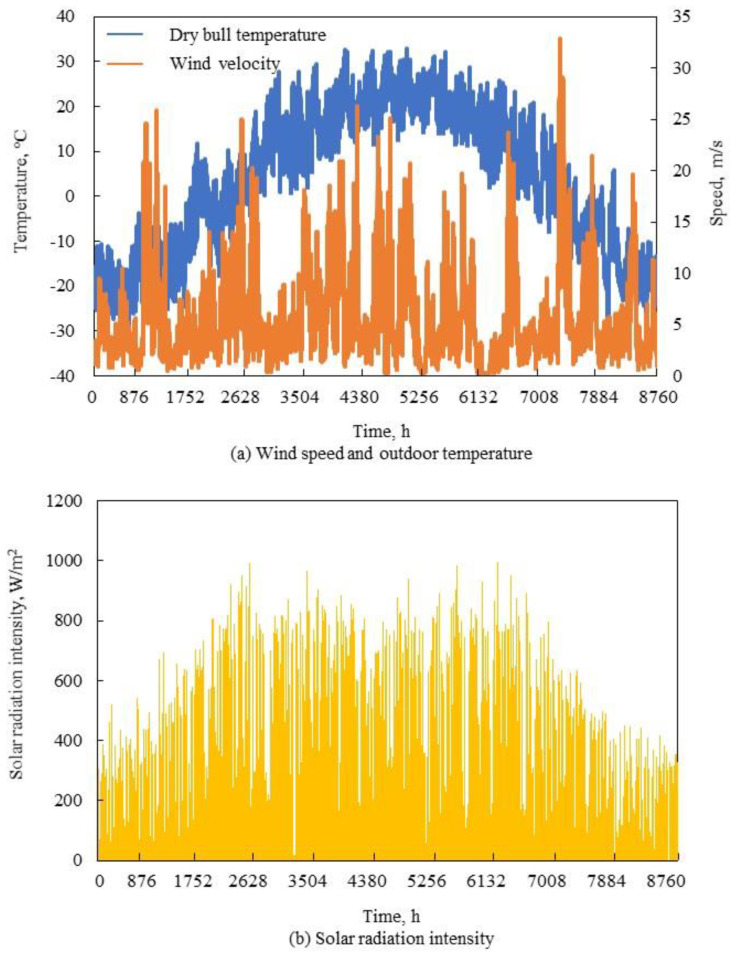
Renewable resource data and outdoor temperature parameters: (**a**) wind speed and outdoor temperature; (**b**) Solar radiation intensity.

Hourly meteorological data—including air temperature, solar radiation and wind speed—were simulated in DeST [27]. The outdoor environment temperature, wind speed, and solar radiation intensity in this area (shown in Figure 4) were suitable for the development of renewable energy technologies.

The technical parameters of the IES and the reference system are listed in Table 2. Table 3 and Table 4 show the initial investment cost of the equipment and key parameters in the optimization solution. The solutions obtained are points on the front of the pareto, using parameters listed in Table 3—and they are evenly distributed in the target space. When the number of calculations reached 300 generations, the optimization results already had a good distribution. Further increase in the number of iterations would not have improved the convergence results. The population size of 100 retained diversity. The mutation probability should be the reciprocal of the number of decision variables. From the theoretical and experimental results [28], the crossover and mutation index was set to 20, and the crossover probability was 0.9—both of which are efficient parameters. The optimization range of the capacity was determined according to the extreme value of the load, and the operating variable was a dimensionless value between 0 and 1.

The influence of different parameters on the results of the optimization algorithm is listed in Table 5. The results showed that a larger population size and iteration number in the parameters of NSGA-II resulted in a longer calculation time. However, when the number of iterations was large, the convergence time was obviously shorter than the calculation time, resulting in unnecessary calculation time. Therefore, selecting the appropriate population size and number of iterations was critical.

### 4.2. Optimization Results

**Figure 5:**
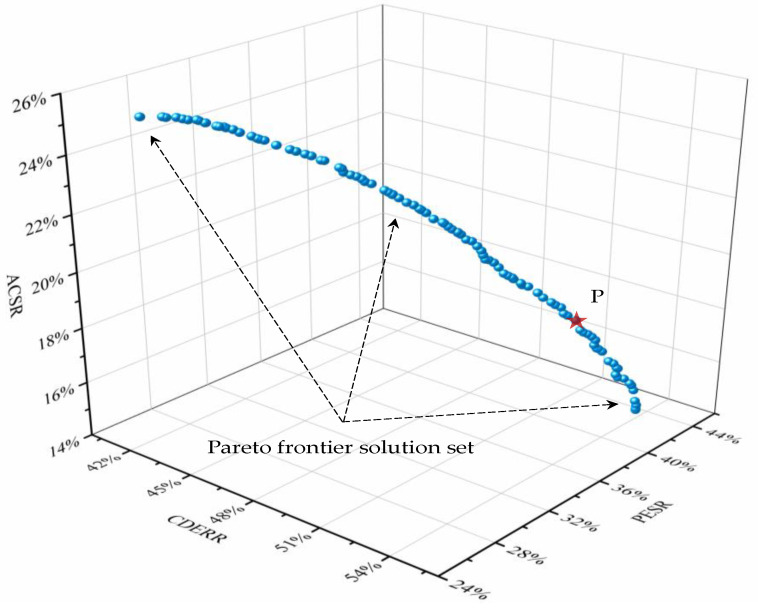
Multi-objective optimization frontier solution set of NSGA-II.

The Pareto optimal solution set was acquired by running the multi-objective optimization algorithm program. The performance indicators of the optimal solution are shown in Figure 5. The value of each coordinate axis represents the performance of the optimization target, and each point is the solution obtained by NSGA-II, which forms the Pareto frontier. The subjective selection solution considers that the environmental, economic and energy efficiency have the same weight, so the point P in the figure with the largest total indicator value was selected as the optimal solution for the performance assessment analysis. After the optimization of capacity configuration and operation strategy, IES achieved performances of ACSR, PESR and CDERR of 17.3%, 39.8% and 53.8%, respectively.

The detailed system technical parameters of the optimal solution point P are listed in Table 6. The total installed capacity of renewable energy generation accounts for 64.5%. The ratio coefficient θ is 0.655, because the initial investment costs of GSHP are lower than that of ACH, and the cooling and heating coefficients of performance are much larger than ACH. As shown in Table 7, the results of different weights (binary weights) of ACSR, PESR, and CDERR are given, so that users can choose different weights for specific applications and scenarios.

### 4.3. Energy Scheduling Analysis

**Figure 6:**
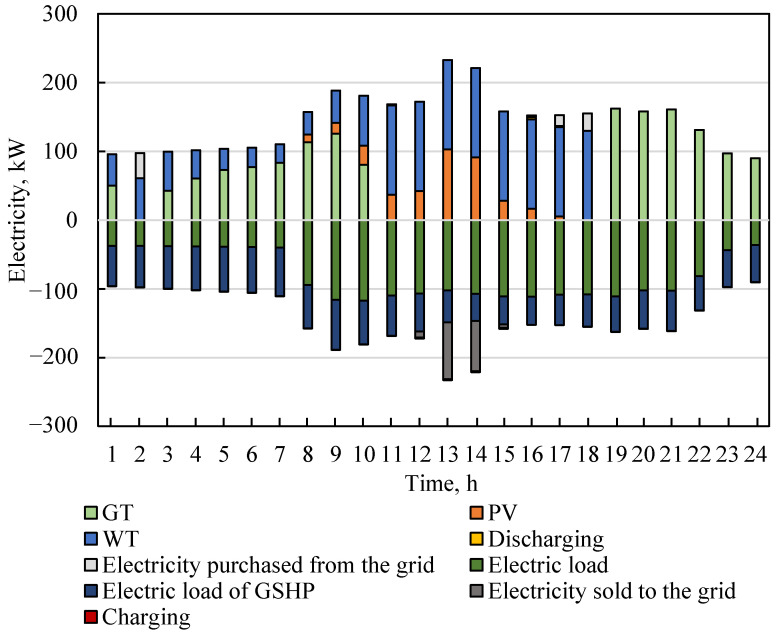
Electricity source and consumption of the integrated energy system (IES) in a typical day.

Power dispatching analyses can clarify the operational status of electrical equipment under optimal configuration and show the interaction between supply and demand. Figure 6 shows the energy dispatch status of the electric source and load demand in a typical day. From 1:00 to 7:00, the electricity generated by the WT was not enough to meet the demand of electric consumption, and photovoltaic electricity generation did not meet the operational conditions; thus, the GT generated electricity under partial load. At the same time, part of the electricity bought from the grid met the electricity load. From 8:00 to 10:00, as the electrical load increased significantly, the power generation of WT, PV and GT jointly met the electrical load. From 11:00 to 18:00, the IES gave priority to the utilization of renewable energy. Due to the low economy and energy efficiency of the GT under low load, the GT was shut down. From 13:00 to 15:00, part of the electricity was stored in the battery, and the surplus was injected into the grid. Conversely, from 16:00 to 18:00, supplementary power was supplied by batteries and the grid. From 19:00 to 24:00, solar radiation and cut-in wind speed could not meet the renewable power generation conditions. Therefore, both the building electrical load and GSHP electrical load were supplied by GT.

**Figure 7:**
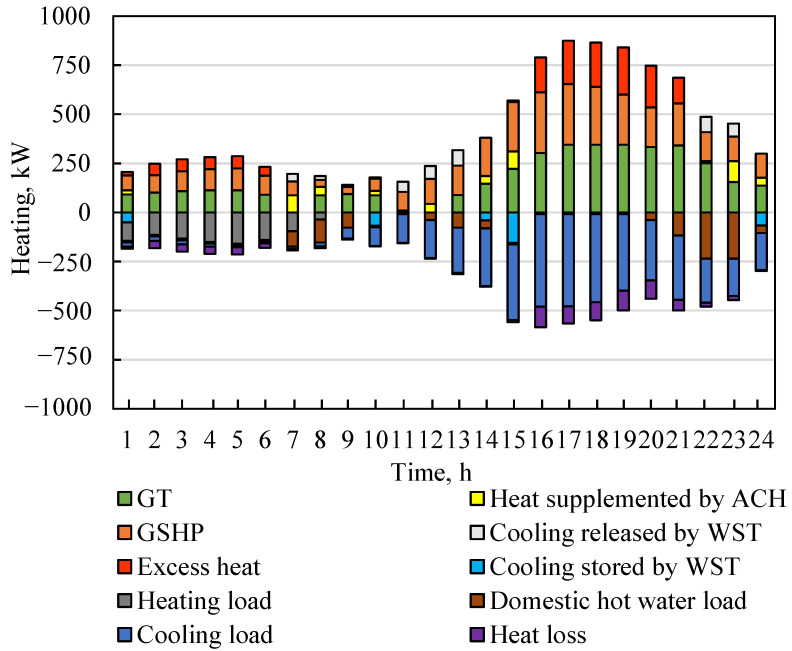
Heating source and consumption of the IES in a typical day.

The heat dispatch shown in Figure 7 reveals the heat source and heat demand in a day. From 1:00 to 6:00, the ambient temperature at night was relatively low. The heating load of the hospital was greater than the cooling load. Part of the chilled water was stored in the WST. However, the total load demand was small, resulting in waste heat generated by the system. From 7:00 to 9:00, ACH directly burned natural gas to drive the cycle to produce domestic hot water to meet the relatively large domestic hot water load. The WST released chilled water, and the ACH and GSHP jointly met the cooling load and heating load. There was no heating water load in the hospital after 10:00. From 10:00 to 15:00, the GT generated less waste heat at a low load rate. Therefore, the GSHP and WST met most of the cooling load. From 16:00 to 21:00, due to the large electrical load, the GT generated a lot of waste heat while generating electricity. Part of the waste heat drove the AC/H to produce domestic hot water and chilled water. However, the cooling capacity of the AC/H accounted for only about 0.34, resulting in a lot of wasted heat. From 22:00 to 24:00, as the waste heat generated by the gas turbine decreased, part of the natural gas entered the ACH to burn and generate heat to drive the circulation to produce domestic hot water. The cooling load demand was relatively reduced, and the insufficient cooling load was met by the WST.

### 4.4. Sensitivity Analysis

**Figure 8:**
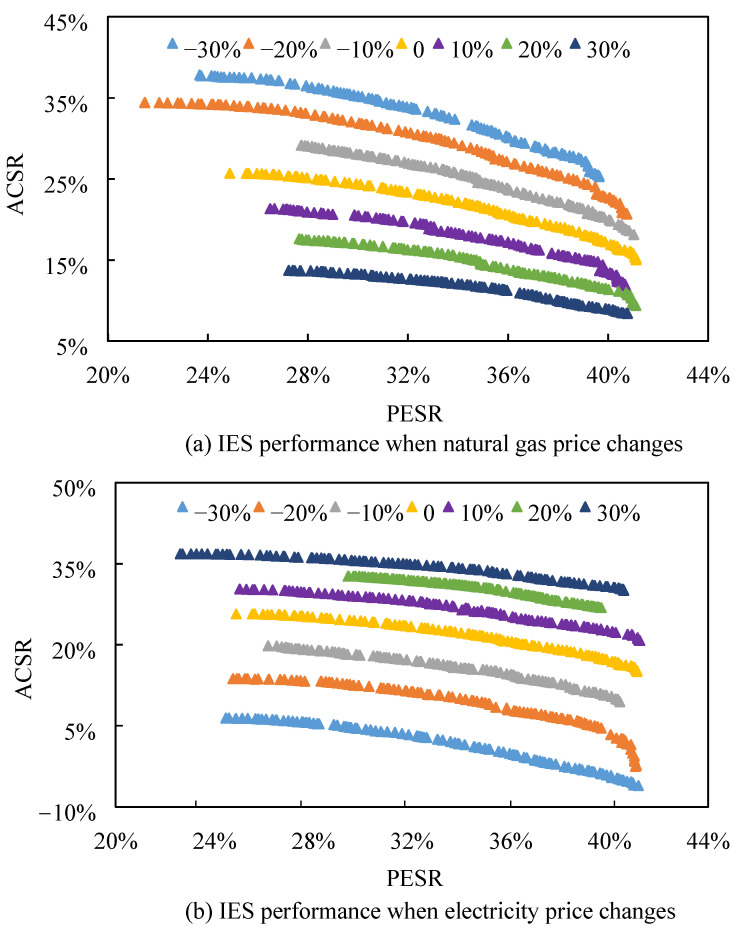
The impact of changes in prices of natural gas (**a**) and electricity (**b**) on IES performance.

Analyzing the effects of energy price changes on system performance is vital for exploring IES. Figure 8a,b shows the direct impact of changes in natural gas prices and electricity prices of the grid on ACSR and PESR. The different colors in Figure 8 represent the prices of energy fuels, which vary from −30% to 30% based on every 10% change. All the points are optimal solutions under different fuel prices. The optimized solution set showed that as natural gas prices increased, ACSR decreased and PESR remained almost unchanged. On the contrary, as natural gas prices increased, ACSR increased, and PESR was practically constant. The reason for this may have been that the evaluation index is a relative value. When the electricity price increases, the cost saving of IES is faster than the cost increases of SP, which leads to an increase in ACSR. However, when natural gas prices increase, the cost savings of IES are slower than the cost increases of SP, which leads to a decrease in ACSR.

**Figure 9:**
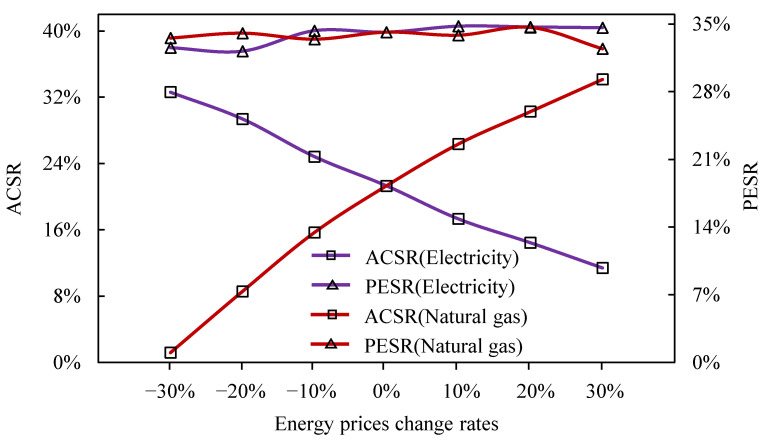
Variations of average ACSR/PESR of the IES with energy price changes.

The detailed changes in the index are shown in Figure 9. The average value of ACSR decreased by 3.5% (and PESR increased by 0.3%) when the natural gas price increased by 10%. On the contrary, the average value of ACSR increased by 5.5%, (and PESR decreased by 0.2%) when the electricity price of the grid increased by 10%. The analysis shows that the economic indicators of the IES were more sensitive to changes in energy prices, and the fluctuation of energy consumption index changes were not obvious.

**Figure 10:**
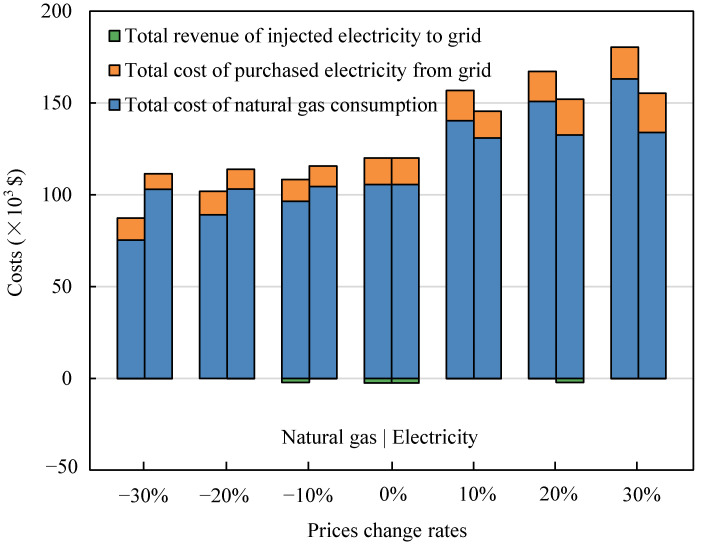
The impact of energy price changes on IES operating costs.

Fuel is the main component of operating cost. The total natural gas consumption costs consist of the fuel cost of the GT and the fuel cost of the AC/H direct combustion zone. Figure 10 shows the impact of energy prices on fuel costs. The results show that the operating cost of IES was more sensitive to the change in natural gas prices compared with the shift in electricity prices. The utilization and distribution of natural gas are critical for the performance of the IES proposed in this paper, and the change in natural gas prices will directly affect the optimization objective.

## 5. Conclusions

This paper proposed a gas-wind-photovoltaic IES, following the establishment of its optimization method for determining the capacities of the devices. Through a case study in a severe cold region, the following conclusions were drawn.

The annual ACSR, PESR and CDERR were incorporated into the multi-objective optimization problem. Their optimal values were 17.3%, 39.8% and 53.8% in the optimal operation strategy. The Pareto frontiers considering ACSR, PESR and CDERR demonstrated that the PESR had a positive relationship with CDERR; it had a negative impact on ACSR.

The optimal capacities of the IES were 167 kW for the GT, 482 kW for the WST, 8 kW for the GT, 173 kW for the PV, 130 for the WT, 406 kW for the ACH and 729 kW for the GSHP. The total installed capacity of renewable energy generation accounted for 64.5%. The optimal capacities—considering different objectives—were variable, and multi-criteria decision-making was necessarily adopted to select a suitable scheme.

Natural gas processes had a more prominent influence on the operating cost of the IES compared with shifts in electricity prices. The utilization and distribution of natural gas is critical for the performance of the IES proposed in this paper, and changes in natural gas prices directly affects the optimization objective.

## Figures and Tables

**Table 1:** Technical parameters involved in the system.

Algorithm Name	Multi-Objective	Advantages	Source
Genetic algorithm	√	Multiple optimal solutions can be obtained and provided by integrating simulation models into optimization tools	[8]
Genetic algorithm and a nonlinear interior point method	×	Hybrid optimization algorithm is more effective for specific model	[13]
Particle swarm optimization	√	The convergence speed is fast	[9]
Artificial neural network	×	It has fault tolerance and can solve nonlinear problems	[10]
Sequential quadratic programming algorithm	×	More effective in solving nonlinear constrained optimization problems	[16]

**Table 2:** Technical parameters involved in the system.

Equipment	Parameter	Value	Source
GT	$\eta_{gt}^{e}$, %	30	[29]
WT	$V_{r}$,m/s	3	[22]
	$V_{ci}$, m/s	12	[22]
	$V_{co}$, m/s	20	[22]
AC/H	$COP_{h,abs}$	0.9	[24]
	$COP_{c,abs}$	1.4	[24]
	$COP_{hw,abs}$	0.9	[24]
	$\eta_{dcz}$, %	80	[23]
GSHP	$COP_{hp}^{c}$	4.3	[30]
	$COP_{hp}^{h}$	5.6	[30]
WST	$\eta_{wst}$ , *%*	90	[23]
PV	$\eta_{e,pv}$, %	16	[23]
	α	−0.5	[23]
	*f*	0.9	[23]
Battery	$\eta_{s}^{e}$, %	96	[29]
Power generation efficiency	$\eta_{p}^{e}$, %	35	[31]
Efficiency of heat exchanger	$\eta_{hx}^{h}$, %	80	[31]
Boiler thermal efficiency	$\eta_{gb}^{h}$, %	80	[31]
Grid transmission efficiency	$\eta_{t}^{e}$, %	92	[31]
Natural gas price	$\delta_{gas}$, kg/kWh	0.220	[31]
Electricity price from grid	$\delta_{grid}$, g/kWh	0.968	[31]

**Table 3:** Technical parameters involved in the system.

System Component	Unit	Initial Investment Costs	Source
GT	$/kW	1046	[23]
WT	$/kW	1196	
PV	$/kW	2039	[23]
ACH	$/kW	225	[23]
GSHP	$/kW	373	[23]
WST	$/kW	56	[23]
Battery	$/kWh	359	[32]
Boiler	$/kW	25	[33]
Heat	$/kW	22	[33]
exchanger			

**Table 4:** The key variable parameters in the optimization solution.

Variable	Value	Variable	Value Range
Population size	100	$N_{gt}$	[0, 180]
Maximum iteration number	300	$N_{pv}$	[0, 180]
Crossover probability	0.90	$N_{wt}$	[0, 180]
Mutation probability	0.16	$N_{s}$	[0, 180]
Distribution index of crossover operator	20	$N_{wst}$	[0, 520]
Distribution index of mutation operator	20	θ	[0, 1]

**Table 5:** The influence of different parameters on the results of the optimization algorithm.

Population Size	Iteration Number	Converge Time (s)	Computational Time (s)
50	100	206.43	240.03
50	300	387.49	687.86
50	500	519.84	1181.45
100	100	454.19	504.66
100	300	923.65	1485.40
100	500	1711.41	2516.78

**Table 6:** Optimization results of IES.

Items	Symbol	IES	SP
GT capacity	$N_{gt}$, kW	167	-
WST capacity	$N_{wst}$, kWh	482	-
Battery capacity	$N_{sc}$, kWh	8	-
PV capacity	$N_{pv}$, kW	173	-
WT capacity	$N_{wt}$, kW	130	-
Proportion of heating and cooling supplied by GSHP	θ	0.655	-
ACH capacity	$N_{ach}$, kW	406	-
GSHP capacity	$N_{gshp}$, kW	729	1113
GB capacity	$N_{gb}$, kW	-	405
HX capacity	$N_{hx}$, kW	-	324

**Table 7:** Optimization results under the binary weight of the indicators.

Symbol	Binary Weights (ACSR, PESR, CDERR)					
	(0,0,1)	(0,1,1)	(0,1,0)	(1,1,0)	(1,0,0)	(1,0,1)
$N_{gt}$, kW	169	169	169	170	177	177
$N_{wst}$, kWh	521	521	521	342	303	342
$N_{sc}$, kWh	21	21	21	0	0	0
$N_{pv}$, kW	180	180	180	179	0	101
$N_{wt}$, kW	180	180	180	95	28	86
θ	0.666	0.666	0.666	0.639	0.582	0.623
$N_{ach}$, kW	425	425	425	405	466	420
$N_{gshp}$, kW	742	742	742	712	648	694
ACSR	15.2%	15.2%	15.2%	18.4%	25.6%	21.7%
PESR	41.1%	41.1%	41.1%	38.8%	24.9%	34.7%
CDERR	54.9%	54.9%	54.9%	53.2%	43.0%	50.3%

## Data Availability

Not applicable.
